# Editorial: Composite materials in biological and bioinspired systems: Part II, Biological and bioinspired composites

**DOI:** 10.1098/rsfs.2024.0016

**Published:** 2024-06-07

**Authors:** Stanislav N. Gorb, Wencke Krings

**Affiliations:** ^1^ Department of Functional Morphology and Biomechanics, Zoological Institute, Christian-Albrechts-Universität zu Kiel, Am Botanischen Garten 1–9, Kiel 24118, Germany; ^2^ Department of Cariology, Endodontology and Periodontology, Universität Leipzig, Liebigstraße 12, Leipzig 04103, Germany; ^3^ Department of Electron Microscopy, Institute of Cell and Systems Biology of Animals, Universität Hamburg, Martin-Luther-King-Platz 3, Hamburg 20146, Germany; ^4^ Department of Mammalogy and Palaeoanthropology, Leibniz Institute for the Analysis of Biodiversity Change, Martin-Luther-King-Platz 3, Hamburg 20146, Germany

**Keywords:** composite, biological, bioinspired

Nature has long been a source of inspiration for scientists and engineers seeking to understand and replicate its remarkable structures and materials for stronger, lighter and more resilient materials. Organisms have spent millions of years perfecting them through the process of evolution, resulting in an array of remarkable biological composites found throughout the six kingdoms of life. These composites, representing a combination of different materials at various length scales, exhibit properties that far exceed those of their individual components. From the sturdy yet lightweight bones of vertebrates to the flexible and tough fibres in spider silk, biological composites are ubiquitous in the natural world, displaying ingenious solutions to structural challenges and are proof of the ingenuity of natural selection and the adaptability of life.

One of the key features of biological composites is their hierarchical structure, which spans multiple length scales. At the nanoscale, they consist of molecular building blocks arranged in specific patterns to optimize mechanical strength and flexibility. These building blocks then assemble into larger structures, such as fibres, lamellae or other compartments, which further enhance the material’s properties through reinforcement. Finally, these structures come together at the macroscopic level to form tissues and organs with exceptional mechanical performance.

The diversity of biological composites is matched only by their versatility. Different organisms have evolved unique solutions to suit their specific habits and environments. For example, the bones of fish fins are optimized for producing large hydrodynamic forces without collapsing, balancing the need for lightweight construction with the demands of mechanical strength. In addition, the scales of fish provide effective protection from predators while remaining flexible enough to withstand the stresses of the ocean environment and support the ability of the body motion.

The study of biological composites not only deepens our understanding of natural systems but also offers valuable insights for the development of biomimetic materials and technologies. By unravelling the design principles behind biological composites, researchers can provide this information to engineers who can create synthetic materials with properties that compete with or even surpass those found in nature for use in aerospace, automotive and construction industries.

In medicine, biomimetic materials offer the potential to revolutionize treatments for a range of conditions. For instance, researchers are exploring the use of synthetic bone grafts that mimic the structure and composition of natural bone to promote healing and regeneration. Similarly, bioinspired adhesives based on the adhesive properties of gecko feet in combination with some advanced chemically based mussel-inspired glues [1] could offer new solutions for wound closure and tissue repair.

Beyond these practical applications, biomimetic materials also hold promise for addressing environmental challenges. By learning from nature’s designs, researchers can develop sustainable materials and manufacturing processes that minimize environmental impact. For example, biobased composites derived from renewable sources offer an eco-friendly alternative to traditional petroleum-based plastics, reducing reliance on fossil fuels and mitigating plastic pollution.

This theme issue explores the properties and applications of biological composite materials, highlighting their importance in addressing current challenges and driving biomimetic innovation in various technological domains.

In the first contribution, Zheng *et al*. [2] investigate the impact of mineralization on collagen matrix structure in turkey leg tendon, using synchrotron X-ray scattering and polarization-resolved second harmonic generation microscopy. The results reveal significant differences in collagen organization and mineral interactions across non-mineralizing, early mineralizing and late mineralizing zones, shedding light on molecular-scale changes in collagen in the presence of mineral deposition and paving the way for future studies into this dynamic process.

In the following study, Taylor *et al*. [3] address the mechanics of split ends in human hair by developing three new laboratory tests to simulate hair tangling during combing. Testing low-quality hair prone to split ends against control hair revealed significant differences, particularly in the moving loop fatigue test where the low-quality hair exhibited fewer cycles before failure. Observations suggest that splits in low-quality hair originate internally and propagate longitudinally, contrasting with surface-originating and short splits in control hair, with implications for future theoretical models of the splitting mechanism.

In Rodrıguez-Ramos *et al*. [4], the asymptotic homogenization method is applied to analyse the mechanical behaviour of periodic multi-laminated micropolar elastic heterogeneous composites under perfect contact conditions. By deriving analytical expressions for effective stiffness and torque coefficients, the effects of rotations in the layers’ constitutive properties on the mechanical response of bi-laminated composites are explored, with implications for bioinspired systems like bone fracture reconstruction using multi-laminated biocomposites, where stability, compatibility and support for cell growth are crucial considerations.

The study by Ghimire *et al*. [5] introduces a novel hierarchical nested honeycomb inspired by energy-absorbing citrus peels, demonstrating distinct energy absorption profiles with two plateaus by integrating secondary hierarchical units. Fine-tuning of these profiles is achieved by adjusting the thickness of primary and secondary cell walls, while a strategic reduction in cell wall thickness at key positions enables material conservation without sacrificing specific energy absorption, offering insights for design optimization in various applications, including packaging and high-speed automobiles, where the nested honeycomb structure exhibits significant improvements in compressive strength and specific energy absorption.

The mechanics of biological composite materials allow for deep knowledge about the interplay between an organism and its environment. In Schulz-Kornas *et al*. [6], the intraspecific dietary variability and biomechanical aspects of chewing in grey wolves from Alaska and Sweden are analysed via three-dimensional surface texture analysis. The wolves from Sweden exhibit rougher surfaces with higher peaks and deeper dales compared with those from Alaska, while males show slightly larger dale area and hill volume than females, suggesting dietary segregation and antagonistic asymmetry in occlusal wear signatures, which provide new insights into the behaviour and ecology of this species.

Liprandi *et al*. [7] explore the mechanical properties of adhesive capture threads in southern house spiders, revealing extreme extensibility. By employing high-resolution mechanical testing and experiments, the hierarchical loops-on-loops structure of the threads is elucidated, highlighting its role in delaying terminal failure and enhancing energy absorption, which offers insights for the bioinspired design of fabrics and materials capable of sustaining high deformation.

The following study by Das *et al*. [8] introduces a synthetic morphing beam inspired by natural fish fins, comprising acrylic outer beams connected by highly compliant rubber ligaments. Through experiments and modelling, it is demonstrated that the ligaments exhibit nonlinear geometrical effects, enhancing the stability of the beam at large deformations while remaining mechanically invisible at small deformations, providing insights for optimizing morphing shape and stiffness in bioinspired structures for diverse applications in aerospace, biomedicine, and robotics.

In Sun *et al*. [9], the application of biological scattering foams and structural coloration principles to manipulate the transmission properties of thermal infrared radiation in polyolefin materials, inspired by chameleons’ skin and everyday technologies like magic water colouring books, is investigated. By engineering microstructured sheets from polyethylene and polypropylene and integrating them with index-matching fluids, they achieved reversible modifications of thermal transmission properties, offering solutions for dynamic thermal management that mimic natural adaptive functionality.

The contribution by Tan *et al*. [10] characterizes elasmoid fish scales, revealing structural and mechanical heterogeneities within individual scales and correlations between the mineralization levels and nanomechanical properties. Finite element modelling demonstrates increased flexibility in some scales, and observations of Mandle’s corpuscles suggest mechanisms of mineral growth and space-filling that enhance the bonding strength between collagen fibre layers.

In Mogas-Soldevila *et al*. [11], an additive manufacturing platform utilizing molecular self-assembly is employed to produce metre-scale structures with complex geometries and heterogeneous material composition, using chitosan, a polysaccharide precursor material. Through a combination of extrusion-based robotic fabrication and directional tool-pathing, the concentration-dependent crystallization and preferred orientation of polymer chains are controlled, leading to anisotropic structures with induced large-scale folding via controlled propagation of residual stresses, as demonstrated and assessed through high-resolution micro-X-ray diffraction and finite element simulations.

The last contribution by Cheng *et al*. [12] explores non-axisymmetric prototypes of artificial adhesive structures to mimic the hair-like attachment structure spatula found in animals, investigating their interactions with rough surfaces using finite element software. The flexibility of the spatula, influenced by its variable thickness, significantly affects contact behaviour, with thinner spatulas showing stronger attachment owing to higher nominal contact area, while thicker spatulas demonstrate regulation of attachment during unidirectional loading, offering new design concepts for biologically inspired adhesives.

As highlighted in this theme issue, the collaborative synergy between biology and materials science in exploring biological composite materials not only drives technological advancement but also enriches our comprehension of organisms shaped by biological evolution. By integrating these disciplines, researchers can drive advancements in both biological understanding and the engineering of materials possessing novel properties and functionalities.

## Data Availability

This article has no additional data.
